# Small Bowel Diverticulosis As a Cause of Chronic Pneumoperitoneum

**DOI:** 10.7759/cureus.7303

**Published:** 2020-03-18

**Authors:** Mark Hanna, Chu Ng, Kellee Slater

**Affiliations:** 1 General Surgery, Princess Alexandra Hospital, Brisbane, AUS; 2 Surgery, University of Queensland, Brisbane, AUS

**Keywords:** small bowel diverticulosis, jejunal diverticulosis, pneumoperitoneum

## Abstract

Pneumoperitoneum, or the accumulation of free air in the peritoneal cavity, is commonly associated with visceral perforation, mandating emergent surgical intervention. Non-surgical pneumoperitoneum, where visceral perforation is not the cause, does not commonly require surgical management. Chronic pneumoperitoneum secondary to small bowel diverticulosis is rare. Of all gastrointestinal diverticular diseases, jejunoileal diverticulosis is the rarest form.

We describe a case of chronic pneumoperitoneum in an 83-year-old male presenting with intermittent abdominal distension and constipation over five years resulting in many presentations to his rural hospital. There were never any associated signs of sepsis such as fever or tachycardia. A computed tomography scan revealed large volume pneumoperitoneum without evidence of perforated viscera or free fluid.

An elective diagnostic laparoscopy revealed extensive small bowel diverticular disease. One of the diverticuli exhibited pneumotosis intestinalis where bubbles of gas were noted within the diverticulum wall and mesentery in the local vicinity. Given the extent of the small bowel diverticular disease, the patient’s advanced age, and relative lack of symptoms, bowel resection was not undertaken and the patient was managed conservatively.

This article illustrates a case of chronic pneumoperitoneum due to small bowel diverticulosis. It highlights the differential diagnoses for chronic pneumoperitoneum, increases awareness of this rare and challenging condition, and portrays the utility of conservative management avoiding major surgery and its potential complications.

## Introduction

Pneumoperitoneum, or the accumulation of free air in the peritoneal cavity, is associated with visceral perforation in 85% to 95% of cases and frequently requires emergent surgical intervention. Non-surgical pneumoperitoneum accounts for 5% to 15% of cases, where free visceral perforation is not the cause, and does not commonly require surgical management [1]. Established causes of non-surgical pneumoperitoneum are classified into thoracic, abdominal, gynaecological, and idiopathic causes [1,2]. Chronic pneumoperitoneum secondary to small bowel diverticulosis is rare. Of all gastrointestinal diverticular diseases, jejunoileal diverticulosis is the rarest form and occurs in approximately 1% to 2% of the population [1,3]. As the clinical presentations of chronic pneumoperitoneum are often non-specific and diverse, diagnosis and management become a challenge [4]. We present a case of chronic pneumoperitoneum secondary to small bowel diverticulosis and discuss conservative management as an appropriate pathway.

## Case presentation

**Figure 1:**
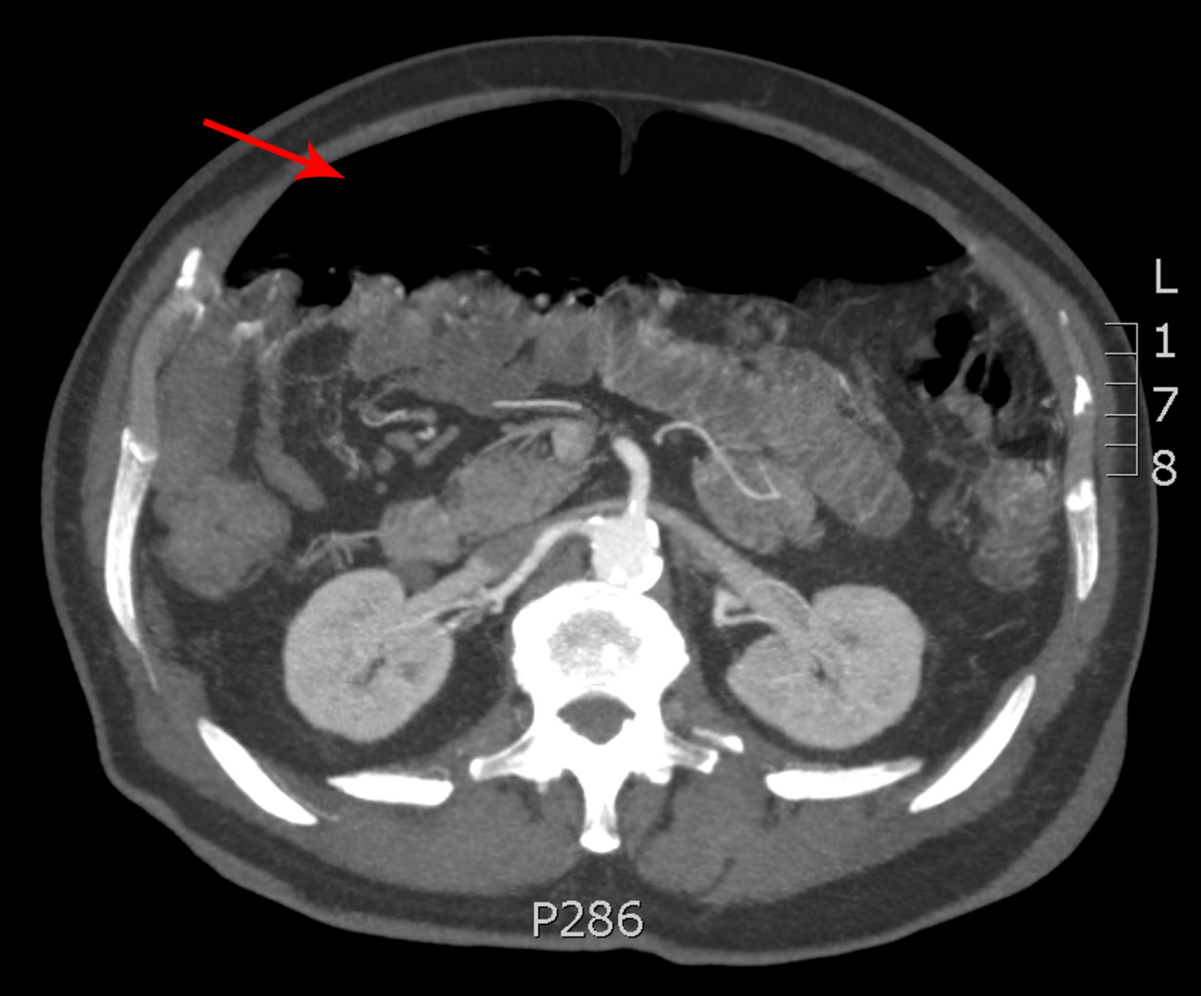
CT scan demonstrating extensive pneumoperitoneum (red arrow)

An 83-year-old male was referred to a tertiary hospital outpatient clinic for opinion and management of his recurrent abdominal distension and constipation over a five-year period. He had multiple similar presentations to his local hospital with no overt clinical signs of sepsis such as fever, pain, or tachycardia to warrant surgical intervention. His past medical history included hypertension, gastro-oesophageal reflux disease, hypothyroidism, dyslipidaemia, and previous inguinal hernia repair. He underwent colonoscopy and gastroscopy which were both normal. On examination, he was haemodynamically stable and in no distress. His abdomen was markedly distended but soft and not tender to palpation. Blood biochemistry revealed normal inflammatory markers. A computed tomography scan revealed significant pneumoperitoneum without evidence of visceral perforation, inflammation, pneumatosis intestinalis or free fluid (Figure 1).

**Figure 2:**
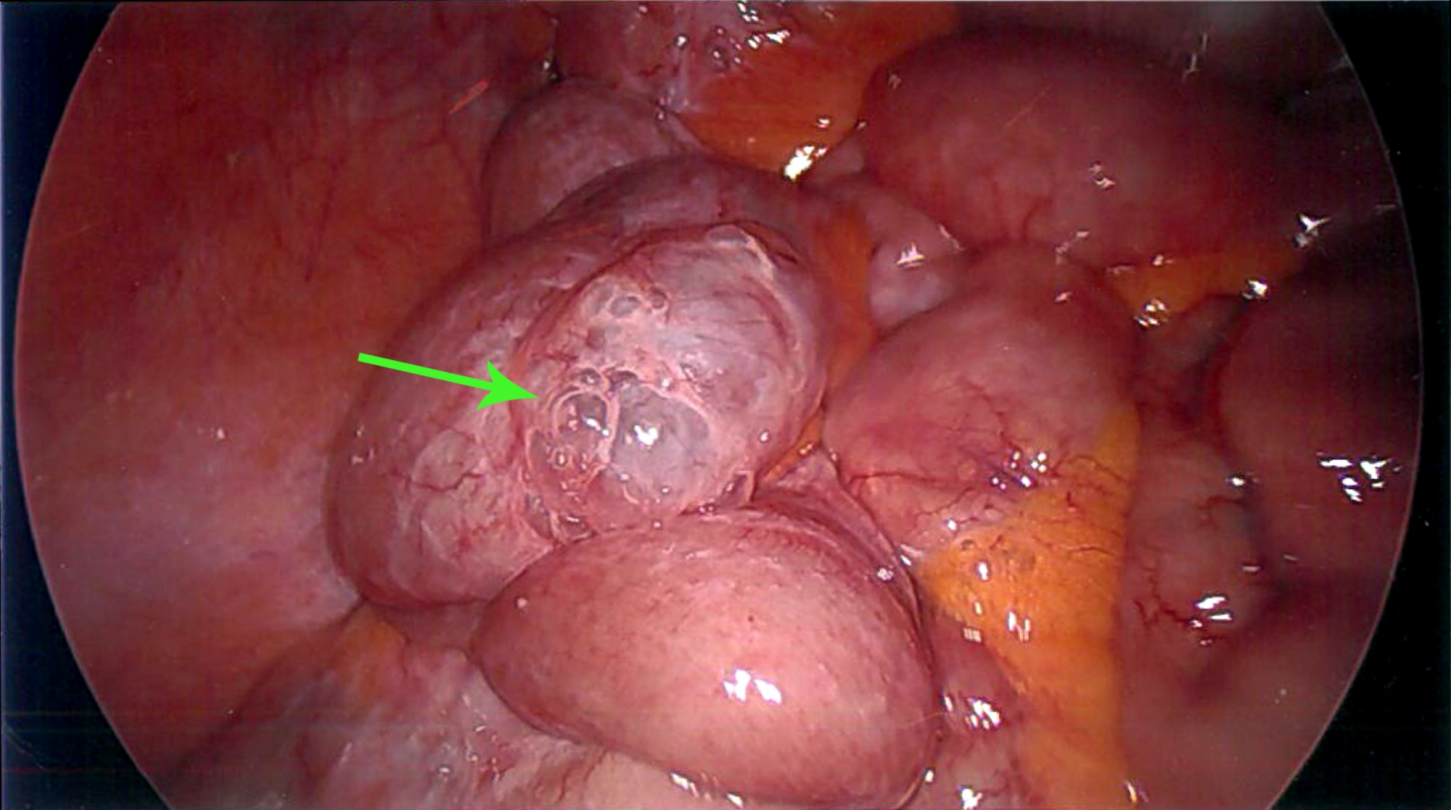
Diagnostic laparoscopy revealing bubbles of gas within the small bowel diverticulum wall (green arrow)

An elective diagnostic laparoscopy to further evaluate the cause of free air was undertaken. There was an audible escape of gas on entering the abdomen and extensive small bowel diverticular disease noted. One of the jejunal diverticuli exhibited pneumotosis intestinalis, where bubbles of gas were noted within the diverticulum wall and the mesentery in the vicinity (Figure 2). Immersing this section of bowel in normal saline failed to demonstrate a leak. There was no evidence of inflammation. A decision was made not to pursue small bowel resection given the increased risk of anastomotic leak due to the extensive diverticulosis, the patient’s advanced age, and relative lack of symptoms. The patient was reassured and his distension was managed conservatively in the community. He is now in his late eighties leading an active life.

## Discussion

Small bowel diverticulosis is rare, occurring approximately in 1%-2% of the population, with 80% of diverticula occurring in the jejunum, 15% in the ileum, and 5% in both [3]. Chronic pneumoperitoneum in the context of jejunal, duodenal, or ileal diverticular disease is uncommon [1,3]. Although most patients with small bowel diverticulosis remain asymptomatic, a small subset of patients, roughly 15% to 20%, experience chronic symptoms [3]. Complications with varying severity may arise including diverticulitis, gastrointestinal haemorrhage, intestinal obstruction, malabsorption, and perforation that may require segmental resection with primary anastomosis [3,5].

In the majority of cases, pneumoperitoneum is secondary to an acute visceral perforation requiring emergent surgical intervention. Chronic pneumoperitoneum, however, is less common and may be managed conservatively in selected cases if peritonitis, fever, and leucocytosis are absent [1,6]. The management of chronic pneumoperitoneum often presents a surgical dilemma due to lack of awareness of this uncommon presentation and vigilance not to miss a potentially detrimental pathology. This case illustrates a typical presentation of chronic pneumoperitoneum with abdominal distension that waxes and wanes in the absence of sepsis where conservative management is appropriate.

The pathophysiology of this type of pneumoperitoneum is not well understood. Dunn and Nelson postulated that the distended diverticular mucosa may function as a semipermeable membrane allowing transmural gas equilibration [7]. According to Longo and Vernava, resection is not recommended except for jejunal diverticula with apparent small bowel loop hypertrophy [3]. Chiu et al. in their review of 88 patients with small bowel diverticular disease concluded that small bowel diverticulosis, with the exception of Meckel's diverticulum, did not need surgical intervention in the absence of significant symptoms [8]. Our patient only had mild chronic symptoms with no signs of sepsis. As the condition was long standing, in an elderly patient, the risk of resection outweighed the benefits and he was managed conservatively after the diagnosis was confirmed.

## Conclusions

Chronic pneumoperitoneum secondary to small bowel diverticulosis is a rare finding and the precise diagnosis may be missed, delayed, or overtreated due to lack of clinical awareness of the disease process. Once diagnosed, it can be successfully treated conservatively by observation and supportive measures alone, avoiding unnecessary laparotomy and potential bowel resection. However, where there is a potential for onset of acute complications, surgical management should be considered.

## References

[1] Mularski RA, Sippel JM, Osborne ML (2000). Pneumoperitoneum: a review of nonsurgical causes. Crit Care Med.

[2] Williams NM, Watkin DF (1997). Spontaneous pneumoperitoneum and other nonsurgical causes of intraperitoneal free gas. Postgrad Med J.

[3] Longo WE, Vernava AM (1992). Clinical implications of jejunoileal diverticular disease. Dis Colon Rectum.

[4] Herrington J (1967). Spontaneous asymptomatic pneumoperitoneum: a complication of jejunal diverticulosis. Am J Surg.

[5] Ferreira-Aparicio FE, Gutiérrez-Vega R, Gálvez-Molina Y, Ontiveros-Nevares P, Athie-Gútierrez C, Montalvo-Javé EE (2012). Diverticular disease of the small bowel. Case Rep Gastroenterol.

[6] Derveaux K, Penninckx F (2003). Recurrent "spontaneous" pneumoperitoneum: a diagnostic and therapeutic dilemma. Acta Chir Belg.

[7] Dunn V, Nelson JA (1979). Jejunal diverticulosis and chronic pneumoperitoneum. Gastrointest Radiol.

[8] Chiu EJ, Shyr YM, Su CH, Wu CW, Lui WY (2000). Diverticular disease of the small bowel. Hepatogastroenterology.

